# Preliminary Education for Medical Students

**Published:** 1883-04

**Authors:** George E. Blackham


### PRELIMINARY EDUCATION FOR MEDICAL STUDENTS.

GEO. E. BLACKHAM, M. D.

In the discussions which have been lately carried on concerning medical education in this country, it seems to be generally admitted,

1st. That a higher grade of education should be required for the degree of M. D.; and,

2d. That this can only be secured by making the course of special medical study longer, and by requiring medical students to have a considerable amount of knowledge at the *beginning* of their medical course, and to prove this by passing a suitable matriculating examination. The chief difference of opinion appears to be as to what should be required of matriculants.

Many able men with the welfare, honor and dignity of their profession at heart, say: "A physician should be a man of culture, of liberal education, he should have taken a classical course before beginning the study of medicine proper. Greek, Latin and Mathematics are absolutely necessary to a liberal education—let us require a fair knowledge of them as a prerequisite for matriculation."

These are desirable accomplishments, doubtless, but, with the possible exception of Latin, by no means necessary.

To determine on what should be the preliminary training of the medical student we must look at the duties of the physician.

What are they? Not, as in former times, to exorcise some demon which has taken possession of the body, or part of it; not to commit to memory complex formulæ of miraculous powders, elixirs of life, and healing salves, but to note in the varying phases of disease the amount and direction of the departure from the normal standards of physiological action, and to so adjust the environment of the patient as to favor a return to physiological action. Disease is no longer looked upon as a malign entity to be met and fought, but simply as a perturbation of function which is to be regulated.

To be able to recognize departures from a standard, we must be familiar with the standard. We must know the details of the operations of the chemical, mechanical and vital forces that keep the human machine in motion for its three score and ten years, more or less. We must also have mind, hands and eyes trained by *practice* to observe, to note trifling differences of temperature, color, expression, etc. No amount of mere book learning, no painful consumption of midnight oil can supply this practical skill, or make up for the want of. As it is now the majority of medical students, even those best prepared, are obliged to get this training after matriculation and, too often, after graduation. How can this observant faculty be trained so that the student may be prepared upon his entrance upon his medical course to receive the full benefit of his clinical opportunities, and at the same time to appreciate the physical, chemical and vital values of the signs and symptoms of diseased action, which his trained power of observation has enabled him to recognize. Remembering that the existence of man as an animal, is three fold, viz: physical, chemical and vital, it is plain that to understand this complex organism it is necessary to study physics, chemistry and biology, in their simpler and less complex manifestations.

To the surgeon some knowledge of mechanics is necessary; to the occulist, optics; to the aurist, accoustics—and can the physician hope to understand the workings of the laboratory in which the complex chemical phenomena of digestion are carried on, oxidizing and deoxidizing, breaking up old compounds here, and making new ones there, without a previous acquaintance with organic chemistry.

How can one expect to understand the vital phenomena of growth, reproduction and death, unless the problems are first studied in their simplest forms? How appreciate the interaction of the myriad cells which go to make up a human body, unless a knowledge of cell life has been obtained in the study of simpler forms of life, in which one cell maintains an independent existence, uninfluenced by partnership with one or more similar or dissimilar cells.

An acquaintance with the principles, at least, of physics, chemistry and biology would seem to be the natural and necessary foundation upon which to build up a secure superstructure of medical knowledge, and it is fortunate that the practical study of these sciences affords the mental training as well as the mere knowledge most needed by the medical student. Looked at merely as mental gymnastics, they are for the practical physician of far more value than all the hoarded treasures of classic lore.

The student of physics, chemistry and biology, who *works* practically at them, who searches out facts for himself, with the aid of scales, lenses, test tubes and dissecting instruments, not only fills his mind with *facts* of the utmost importance to him, as a physician, or surgeon, but at the same time trains hand, and eye, and brain in what must be the business of his life.

No mere parrot-like learning by rote can avail him; he learns to observe, record and reflect; his touch becomes delicate, his eye and ear acute and accurate, and his mind receptive.

The student then who enters upon his medical course with a reasonable practical knowledge of physics, chemistry and biology, who has familiarized himself with the use of the test-tube, the microscope and the dissecting knife, has already mastered the alphabet of his profession, and is prepared to profit by the lectures in the amphitheatre, the demonstrations in the dissecting room, and the clinics in the hospitals in a higher degree than he could by any other course of preliminary training, and is as far in advance, in a practical point of view, of his fellow student with a merely classical education, as he, in his turn, is of the mere ignoramus.

If time would permit, doubtless it would be well for the student to take *both* the scientific and classical courses before entering upon his medical course proper, but, unfortunately, but few medical students can afford the time for this.

Our medical colleges should not either matriculate or graduate ignorâmuses, nor should any part of the brief three years of study be devoted to the mere alphabet of medical science. Let the colleges then insist upon a certain amount of preliminary education, but let it be modern rather than ancient, scientific rather than classical, practical rather than theoretical.

Let the student *demonstrate* his ability to manage test tubes, retorts and re-agents, the miscroscope, the section cutter and the scalpel, and let this practical knowledge rank, at least, as high as the ability to construe Latin or Greek, or work out problems in the calculus; nay, let it rank higher, as equally useful for culture and far more useful for practical purposes.

When such practical tests are made the prerequisites for matriculation at our medical colleges, the faculty will find themselves at the head of a body of students already trained in habits of observation, already familiar with the basic phenomena of life, ready to follow them into the hospital ward, the dead house and the dissecting room; ready and able to see, to recognize and to assimilate the facts before them, and prepared to hear and to understand the teachings of the lecture room, instead of, as is now too often the case, a set of young men who, from lack of training, have not the ability to see, to hear or to understand, and whose first year at medical college is often spent, not in learning, but in *learning to learn.*

To urge forward this reform, to strive for the triumph of practical science, to drive out ignorance, and to encourage individual investigation, should be the work of every lover of his profession. To engage in it is to do more for the elevation of the profession, and the preservation and increase of its dignity than can be done by passing the most rigorously exclusive codes of ethics.

Large bodies move slowly, and reform is a plant of deliberate growth. It can not be expected that either societies or colleges will be in a hurry to enforce so radical a change, but we can each do something. If nothing more, we can see to it that no student goes to medical college from our offices, or with our endorsement, till he has a fair knowledge of the principles of chemistry and physics, and knows enough of practical biology to enable him to understand at once the lectures he is to hear.

By insisting upon this rule we may diminish the number of medical students, but, if statistics can be depended upon, that will not be an unendurable calamity, and what is lost in quantity will be more than made up by the superior quality. Suppose we try the experiment.

